# Changes in the Paradigm of Traditional Exercise in Obesity Therapy and Application of a New Exercise Modality: A Narrative Review Article

**Published:** 2019-08

**Authors:** Hun-Young PARK, Won-Sang JUNG, Jisu KIM, Hyejung HWANG, Kiwon LIM

**Affiliations:** 1. Physical Activity and Performance Institute (PAPI), Konkuk University, Seoul, Republic of Korea; 2. Department of Physical Education, Konkuk University, Seoul, Republic of Korea

**Keywords:** Obesity, High-intensity interval training, Whole-body vibration training, Hypoxic therapy

## Abstract

**Background:**

Obesity is recognized as an important global health problem that increases the risk of all-cause death. It is a major risk factor for various cardiovascular and metabolic diseases.

**Methods:**

We conducted this review through searching the related literature plus internet links.

**Results:**

Recently, many researchers have been applying various efficient alternative exercise paradigms for treating obesity, such as high-intensity interval training, whole-body vibration training, and hypoxic therapy. Compared with moderate-intensity continuous training, high-intensity interval training involves a shorter exercise time but higher energy expenditure and excess post-exercise oxygen consumption via a higher exercise intensity and is effective for treating obesity. Whole-body vibration training effectively reduces the rate of fat production and accumulation through passive vibration of the whole body and improving the body composition, muscle function, and cardiovascular function of the obese population. Hypoxic therapy has been reported to improve obesity and obesity-related diseases through appetite loss, reduced dietary intake, increased energy consumption, improved glycogen storage and fatty acid oxidation, angiogenesis and left ventricle remodeling, decreased mechanical load, and reduced sarcopenia progression due to aging.

**Conclusion:**

The new therapeutic exercise modalities, namely, high-intensity interval training, whole-body vibration training, and hypoxic therapy, are practical, useful, and effective for improving obesity and various metabolic and cardiovascular diseases induced by obesity.

## Introduction

Obesity is caused by the accumulation of excessive body fat via a positive energy balance, manifested by a higher energy intake than energy consumption during resting, physical activity, and exercise (1). Obesity is recognized as an important global health problem that increases the risk of death from all causes and is a major risk factor for various cardiovascular and metabolic diseases (2, 3).

Among the various methods of treating obesity, physical activity is effective in treating obesity via weight and visceral fat loss and for maintaining or increasing muscle mass (4–7). The traditional exercise modality for obesity treatment is moderate-intensity continuous training (MICT), which involves continuous exercise at a moderate intensity, without rest, for at least 30 minutes (8). High-intensity interval training (HIIT) is an exercise modality involving repeated performance of high-intensity exercises and short periods of rest. Currently, HIIT is widely used as an efficient alternative to the traditional exercise modality because of its high energy consumption and excess post-exercise oxygen consumption (EPOC) relative to those during exercise (9–11). Whole-body vibration training (WBVT), which uses equipment to manually vibrate the whole body, is also widely used as a new form of exercise for treating obesity (12, 13). The application of WBVT in an obese population for more than 6 weeks has been reported to reduce the rate of fat production and accumulation and to improve body composition, neuromuscular function, and cardiovascular function (14–19). In addition, hypoxic therapy, which involves various forms of intermittent exercise under hypoxic conditions, is widely used as a new exercise paradigm for obesity treatment in many countries (1, 20, 21). Hypoxic therapy has been reported to improve obesity and obesity-related diseases via appetite loss, reduced dietary intake, increased energy consumption, improved glycogen storage and fatty acid oxidation, angiogenesis and left ventricle remodeling, decreased mechanical load, and reduced sarcopenia progression due to aging (1, 21–24).

This review summarizes the recent evidence that suggests that the new exercise paradigms, namely HIIT, WBVT, and hypoxic therapy, can be valuable and viable therapeutic modalities for obesity and obesity-related diseases.

### Effectiveness of HIIT for treating obesity

Until recently, the most commonly used exercise modality for obesity treatment has been MICT (8). Currently, HIIT is being widely used for obesity treatment (9, 10). HIIT is popular for treating obesity because of its high energy consumption and EPOC over the exercise time (11). HIIT has been reported to improve obesity and obesity-related diseases by reducing abdominal visceral fat, maintaining or increasing fat mass, and improving metabolic function (insulin sensitivity) through greater stimulation of skeletal muscles (8, 22–24).

Wewege et al (8) examined 13 previous studies that compared MICT and HIIT and reported an exercise frequency of 3.3 ± 0.7 times per week for both MICT and HIIT and an exercise duration of 158.3 ± 43.0 minutes/week for MICT and 95.2 ± 46.3 minutes/week for HIIT. According to a meta-analysis, both MICT and HIIT that involved running exercise for more than 10 weeks resulted in a similar reduction in body weight (approximately 2 kg), body fat (approximately 6%), and waist circumference (approximately 3 cm). However, HIIT required a 40% shorter exercise time than MICT. Shuster et al. (23) reported that a 12-week exercise program reduced abdominal visceral fat by 11.1% and 19.5% in the MICT and HIIT groups, respectively. Zhang et al (24) examined the usefulness of MICT and HIIT for reducing general and abdominal fats by using computed tomography in 45 overweight women. They reported that MICT and HIIT showed the same proportions of changes in total body fat and blood lipid levels. Abdominal subcutaneous fat decreased in both groups, but HIIT showed a larger decrease, and abdominal visceral fat showed a significant decrease only in the HIIT group (18% vs. 8% for the MICT group). Maillard et al (22) verified the effect of MICT and HIIT for 16 weeks (twice a week) on reducing general and abdominal fats in 17 menopausal women with type 2 diabetes mellitus. They found no significant difference in serum lipid levels and diabetes mellitus between the MICT and HIIT groups, but significant decreases in abdominal (−8.3%) and visceral (−24.2%) fat percentages were observed in the HIIT group.

Taken together, these previous studies showed that HIIT is a time-efficient and sustainable strategy for improving body composition. In general, for many people, the lack of time can be a barrier resulting in a negative attitude toward exercise. Thus, high-intensity exercise programs, such as HIIT, which are less time consuming, can be offered as options for treating obesity in various people (8, 25). Rognmo et al (26) also reported that HIIT does not increase the risk of adverse events in high-risk groups such as those with coronary artery disease. These results suggest that HIIT may be useful as a sustainable and long-term intervention for many overweight or obese people who spend less time on exercise training. In the future, it will be necessary to study various changes in fat mass in the whole body and local areas using methods such as computed tomography, magnetic resonance imaging, and dual-energy x-ray absorptiometry, which have a higher validity and reliability for measuring body fat. However, when HIIT is used for patients with severe obesity, a more careful approach is needed because mechanical stress may damage joints.

### Effectiveness of passive exercise for treating obesity

The most commonly used passive exercise method for treating obesity is WBVT, which passively vibrates the whole body. As the name implies, WBVT produces motion by applying vibrational stimulation to the whole body in different postures on a vibrating platform. Generally, WBVT equipment generates vibration using rotation or vertical stimulation, which provides an exercise effect. Initially, WBVT was used primarily as an alternative form of exercise for resistance training (13). WBVT mechanically induces rapid changes in the length of the muscle-tendon complex resulting in repetitive eccentric-concentric muscle movement and reflexive muscle contraction (27, 28). In other words, WBVT is universally recognized as an alternative to resistance exercise to improve muscle activity, strength, and power associated with muscle training (12, 29), and is used to improve the exercise performance of athletes and young adults (27, 30, 31).

Recently, WBVT has been reported to reduce body fat in overweight and obese people by reducing the rate of fat production and accumulation, and improve bone density, muscle strength, and cardiovascular function (14–19). WBVT for 10 weeks has been reported to induce significant weight loss in overweight and obese individuals (17, 32–36). Even if weight is not reduced, WBVT induces body composition remodeling through reduction of body and visceral fat percentages (33, 36–40). In addition, WBVT stimulates white adipose tissue through increased central sympathetic activity, increases lipolysis, improves insulin action and glucose control to improve blood glucose control, and promotes the release of growth hormone and metabolic stimulation, which leads to a decrease in body fat (13, 41–45). These mechanisms are reported to be effective in postmenopausal women, especially those with increased insulin resistance and decreased bone density due to hormonal changes (46, 47).

Visssers et al (36) examined the effects of 6 months of WBVT on body weight and visceral fat in comparison with those of aerobic exercise in 61 overweight and obese individuals. They reported that both exercise types were effective in reducing body weight and visceral fat; however, the reduction in body weight and visceral fat tended to be larger for WBVT than for aerobic exercise. Bellia et al (41) examined the effects of WBVT for 8 weeks on body composition and insulin resistance in 34 obese patients divided into a control group (low-calorie diet) and an experimental group (low-calorie diet plus WBVT). The experimental group showed a larger weight loss and greater improvement in insulin sensitivity and adiponectin levels than the control group. Sañudo et al (35) studied the effects of WBVT for 12 weeks on leg blood flow and body composition in 40 patients with type 2 diabetes divided into a control group and a WBVT group. WBVT showed significant improvement in body composition (e.g., body weight, waist circumference, waist-hip ratio, and percent body fat) and increased insulin-mediated glucose uptake in skeletal muscles due to increased blood flow in the lower femoral artery. They suggested that WBVT could be an effective means of increasing leg blood flow and reducing obesity in patients with type 2 diabetes. Figueroa et al (37) verified that 6 weeks of WBVT improved arterial function, blood pressure, sympathovagal balance, and muscle strength in a crossover study of 10 obese women in their 20s. In addition, fluid movement induced by WBVT has been reported to increase bone density by stimulating osteocytes and osteoblasts by creating shear stress in the plasma membrane (13, 19).

Many of these studies support the claim that WBVT is a highly effective method of promoting health in a wide range of subjects, including obese patients who cannot perform traditional exercise (e.g., MICT). In conclusion, WBVT is an effective alternative exercise modality that prevents and improves obesity and obesity-related diseases with less burden on the back, waist, and knee joints because it generates less mechanical stress than traditional exercise methods such as MICT and HIIT. However, the use of WBVT for therapeutic purposes is still not standardized, and its potential adverse effects are uncertain (13, 40). Especially the effects of long-term vibration stimulation on the human brain have not been studied (13). Therefore, standardization is needed for the therapeutic use of WBVT in the management of obesity, and a precise description of the causal relationship between vibration parameters (amplitude, vibration type, and vibration speed) and the results is also needed.

### Effectiveness of hypoxic therapy for treating obesity

Exercise modalities such as MICT, HIIT, and WBVT usually require at least 12 weeks of exercise intervention for prevention and treatment of obesity, but they have the disadvantage of increasing appetite and dietary intake due to sustained exercise (1, 48). On the basis of previous studies that showed that people living at high altitudes have lower percent body fat, blood lipid levels, arterial pressures, and prevalence rates of obesity-related disease than people living at sea level, many advanced countries are using hypoxic condition as a new therapeutic modality for obesity (1, 21, 49). Exercise intervention under hypoxic conditions, owing to various physiological mechanisms, has been reported to reduce appetite and dietary intake, increase blood flow in muscle tissue, increase energy consumption, reduce mechanical load, improve glycogen storage and fat oxidation, slow sarcopenia due to aging, and induce angiogenesis and left ventricle remodeling (1, 21, 50–53).

Hypoxia is a state of reduced oxygen supply to tissues due to decreased oxygen saturation of arterial blood (54). Living long-term at a high altitude has been reported to reduce the possibility of obesity (55), and short-term stays for 1–3 weeks at a high altitude reduce weight and arterial pressure and improve metabolic function without exercise intervention (56–63). However, it is impossible for obese people to live in a natural high altitude, which may cause side effects such as obstructive sleep apnea or acute mountain sickness (63, 64).

**Fig. 1:**
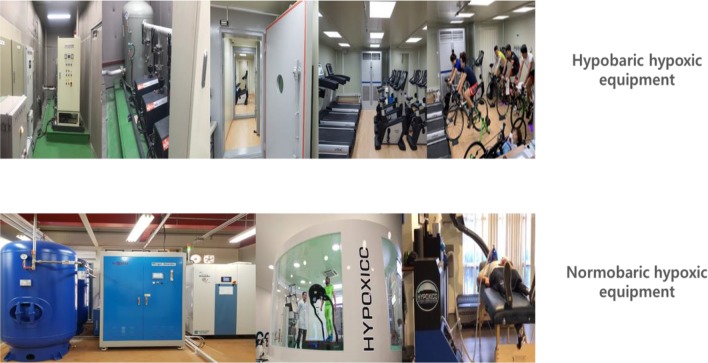
Hypobaric and normobaric hypoxic equipment

Therefore, various types of commercial equipment are being developed to artificially create hypobaric hypoxia and normobaric hypoxia (65). Hypobaric hypoxia is a hypoxic condition created by using a vacuum pump to reduce the air pressure in a rigid structure that can withstand high-pressure differentials. This method is difficult to apply to obese patients because it is inconvenient and expensive. Normobaric hypoxia is a hypoxic condition created by lowering the relative oxygen concentration through nitrogen injection using a nitrogen generator, which is inexpensive and simple to use and be can easily applied to the obese population (Fig. 1). For normobaric hypoxia, the subject is exposed to a hypoxic condition with a 15.0–12.0% fraction of inspired oxygen (F_I_O_2_), simulating an altitude of 2,600–4,300 m, while breathing using a mask or staying in a specific space such as a chamber, room, or tent (50).

Workman and Basset (66) examined the effects of 3 hours of acute hypoxia exposure (hypoxic condition of approximately 80% peripheral capillary oxygen saturation) daily for 7 days on energy expenditure and substrate utilization in 11 active overweight men. Acute exposure for 3 hours a day and intermittent exposure for 7 days to hypoxic conditions increased energy expenditure and lipid oxidation in active overweight men, indicating that exposure to hypoxic conditions enhances metabolic adaptation for obesity treatment. Exposure to hypoxic conditions simulating an altitude of 2,500–3,500 m for approximately 10 days decreased body weight and percent body fat via glucagon-like peptide-1 inhibition, improved insulin sensitivity, decreased dietary intake and serum leptin level elevation, increased basal metabolic rate, and decreased diastolic blood pressure, thereby effectively preventing and treating obesity and obesity-related diseases (57, 67). Exercise intervention in hypoxic conditions has been reported to improve obesity and obesity-associated complications through activation of the body’s immune function, strengthening metabolic function, reducing body weight and percent body fat, and strengthening the cardiovascular system, even though it lowers exercise and mechanical stress (1, 20). Especially exercise intervention in hypoxic conditions (simulating an altitude of 2,000–3,000 m) induces specific molecular adaptations that do not occur during exercise in normoxia (68–72).

Specific molecular adaptation via exercise intervention in hypoxia is associated with increased secretion of norepinephrine (73), decreased plasma leptin levels (74, 75), decreased appetite (1, 76), increased number of mitochondria (21), increased glycolytic enzyme activity (77), increased insulin sensitivity, and decreased diastolic blood pressure (52, 78). The metabolic phenotype of obese subjects seems to be improved by these physiological adaptations. In a previous study, Haufe et al (79) divided 20 healthy men into two groups that performed similar exercise training at 15% F_I_O_2_ and 21% F_I_O_2_; all subjects exercised three times weekly for 60 min for 4 weeks at a heart rate measured at 3 mmol/L lactate at pretraining exercise testing. They reported greater improvements in percent body fat, triglycerol and fasting insulin levels, area under the curve for insulin levels, and homeostasis model assessment index during an oral glucose tolerance test, despite the fact that the absolute exercise load under hypoxic conditions was lower than that under normoxic conditions (hypoxia vs normoxia = 1.4 vs. 1.7 watts/kg). Wisner et al (72) performed a single-blind study to examine the hypothesis that exercise intervention in hypoxic conditions (n = 24, F_I_O_2_ = 15%) would more greatly improve body weight loss and metabolic risk-related parameters than exercise training in normoxic conditions (n = 21, F_I_O_2_ = 21%). They reported that exercise training in hypoxic conditions induced greater improvements in health-related fitness, body composition, and metabolic risk-related parameters despite lower absolute exercise loads than those in normoxic conditions. Kong et al (69) randomly assigned subjects to either a normobaric hypoxia (F_I_O_2_ = 16.4–14.5%) or a normobaric (F_I_O_2_ = 21%) training group, and all the subjects experienced 16 hours normoxia and 6 hours hypoxia or 22 hours normoxic training weekly. They investigated whether exercise intervention in normobaric hypoxic conditions combined with a low-caloric diet had an additive effect on weight loss as compared with normoxic training in obese young adults. Exercise intervention in hypoxia with a low-caloric diet additively improved body weight loss as compared with normoxic training (−6.9 kg or −7.0% vs. −4.3 kg or −4.2%). Park and Lim (52) determined the effect of aerobic exercise in hypoxic conditions for 6 weeks on body composition, blood pressure, arterial stiffness, and blood lipid levels in 35 obese women aged 30–60 years with a body mass index >30 kg/m^2^ and 30% body fat. The participants were divided into three training groups, as follows: normoxic, 16.5% O_2_ hypoxic, and 14.5% O_2_ hypoxic. They demonstrated that exercise training under hypoxic conditions had positive effects on body composition, blood pressure, arterial stiffness, and blood lipid levels in middle-aged obese women as compared with exercise intervention in normoxic conditions.

Given the biomechanical aspects, hypoxic conditions can meet the metabolic demands for obesity prevention and treatment at a lower exercise intensity than normoxic conditions. In other words, exercise in hypoxic conditions involves lower mechanical stress than normoxic conditions but results in greater weight loss and benefits for metabolic and cardiovascular health. Thus, exercise in hypoxic conditions is a highly effective exercise modality that reduces the risk of damage to the musculoskeletal system in obese subjects and allows them to reach their target energy consumption (20). Furthermore, resistance exercise under hypoxic conditions seems to more effectively prevent sarcopenia, which progresses rapidly with aging and obesity, and is highly correlated with cardiovascular and metabolic diseases, via greater hypertrophy, increased muscle strength and endurance, and increased angiogenesis in skeletal muscles as compared to those under normoxic conditions (21, 51, 80).

Taken together, combined exercise intervention and hypoxic conditions are considered effective in preventing and treating obesity and obesity-related diseases by improving various physiological mechanisms and preventing injury through lower mechanical stress and exercise intensity.

## Conclusion

To date, the most common exercise modality for preventing and treating obesity has been MICT, which is a traditional exercise method involving continuous exercise at a moderate intensity without resting for at least 30 minutes. Currently, various exercise programs such as HIIT, WBVT, and hypoxic therapy, which can reduce body weight and improve the health of obese subjects more efficiently, in accordance with the demands of the current lifestyles, are being developed. HIIT can be effective for people with limited time because of its higher energy consumption than MICT. WBVT is a form of passive exercise that can improve muscle function and bone density through muscle nerve stimulation and weight loss in obese subjects. Finally, hypoxic therapy is a new exercise modality that can prevent and treat obesity and obesity-related disease via lower mechanical stress and various physiological mechanisms such as reduced dietary intake and appetite, improved body composition, and enhanced metabolism and cardiovascular fitness.

## Ethical considerations

Ethical issues (Including plagiarism, informed consent, misconduct, data fabrication and/or falsification, double publication and/or submission, redundancy, etc.) have been completely observed by the authors.
